# High Prevalence of Cervical High-Risk Human Papillomavirus Harboring Atypical Genotypes in Human Immunodeficiency Virus -Infected and -Uninfected First-Generation Adult Immigrant Women Originating from Sub-Saharan Africa and Living in France

**DOI:** 10.1007/s10903-020-01074-7

**Published:** 2020-08-20

**Authors:** Ralph-Sydney Mboumba Bouassa, Camelia Gubavu, David Veyer, Leman Robin, Anne Gravier, Laurent Hocqueloux, Thierry Prazuck, Hélène Péré, Laurent Bélec, for the ANRS ImmiPap Study Group, C. Gubavu, A. Gravier, L. Hocqueloux, T. Prazuck, O. Patey, O. Bouchaud, L. Traore, M.K. Youssouf, L. Bélec, R.-S. Mboumba Bouassa, H. Péré, D. Veyer

**Affiliations:** 1 Laboratoire de Virologie, Hôpital Européen Georges Pompidou, Paris, France; 2 Faculté de Médecine Paris Descartes, Université Paris Descartes (Paris V), Sorbonne Paris Cité, Paris, France; 3 École Doctorale en Infectiologie Tropicale, Franceville, Gabon; 4 INSERM U970, Paris Cardiovascular Research Centre, Université Paris-Descartes, Sorbonne Paris Cité, Hôpital Européen Georges Pompidou, Paris, France; 5 Service Des Maladies Infectieuses Et Tropicales, Centre Hospitalier Régional D’Orléans, La Source, France

**Keywords:** Human papillomavirus, First-generation, Immigrant women, Sub-saharan africa, France, Human immunodeficiency virus, Cervical cancer

## Abstract

Human papillomavirus (HPV)-related cervical lesions in first-generation immigrant African women in France should reflect the epidemiology of high-risk (HR)-human papillomavirus (HPV) infection in sub-Saharan Africa. First-generation immigrant African women attending the *Centre Hospitalier Régional* of Orléans, France, were prospectively subjected to endocervical swabs for HPV DNA PCR and Pap smear. Fifty women (mean age, 41.7 years) living in France (mean stay, 10.7 years) were enrolled, including 26.0% of HIV-negative women from general population and 74.0% of women with known HIV infection. Cervical HPV prevalence was 68.0%, with 56.0% of HR-HPV. HR-HPV -68 and -58 were the predominant genotypes (20.0% and 14.0%, respectively). HR-HPV-16 and HR-HPV-18 were infrequently detected. HIV-infected women showed a trend to be more frequently infected by HPV than HIV-negative women (70.3% *versus* 61.5%). Most women (84.0%) showed normal cytology, while the remaining (16.0%) exhibited cervical abnormalities and were frequently HIV-infected (87.5%). These observations highlight the unsuspected high burden of cervical HR-HPV infections mostly associated with atypical genotypes, HIV infection and cervical abnormalities in first-generation immigrant African women living in France.

**Electronic supplementary material:**

The online version of this article (10.1007/s10903-020-01074-7) contains supplementary material, which is available to authorized users.

## Introduction

High-risk (HR)-human papillomavirus (HPV) genotypes are responsible for 5.2% of all cancers worldwide, 2.2% of cancers in developed countries and 7.7% of all cancers in developing countries [1–3]. In sub-Saharan Africa, cervical cancer associated with persistent cervical HR-HPV infection is the most common cancer in women in many countries, with more than 75,000 new cases and nearly 50,000 deaths occurring each year [4, 5]. According to the World Health Organization, cervical cancer will kill annually about half of a million women by the next decade, mostly in Sub-Saharan Africa where HIV epidemic and other risk factors are worsening the burden of this cancer [5, 6]. Thus, cervical cancer has become progressively one of the main public health challenges to overcome in sub-Saharan Africa in recent decades [7].

Sub-Saharan African adult female population harbors high rates of cervical HR-HPV infections that can reach 46.2% in HIV-negative and 79.1% in HIV-positive women, with frequent variations from one region to another [8–10]. Likewise, there is a wide heterogeneity in the distribution of circulating HR-HPV genotypes throughout the African continent [10]. The high-risk genotypes targeted by the Gardasil-9® vaccine (HPV-16, -18, -31, -33, -45, -52, -58), and also non-vaccine high-risk types such as HPV-35, HPV-68 and HPV-59 are among the most detected genotypes both in African women with normal cytology as well as in those with low and high-grade cervical lesions and cancers [8–11].

Around 300,000 immigrant women originating from sub-Saharan Africa are living in France [12]. Most first-generation African immigrant women arrive in France at the beginning of their adult life and most of them arrive already sexually active [12]. First-generation immigrant African women living in France could harbor an infectious profile reflecting the epidemiology of their country of origin where cervical HR-HPV infection is highly prevalent and exacerbated by the so-called “syndemic” synergy played by HIV epidemic and other sexually transmitted infections (STI) [13].

The aim of the present cross-sectional study was to assess the prevalence and genotypes distribution of cervical HR-HPV infection and associated risk factors in HIV-positive and HIV-negative immigrant African women living in France and attending the *Centre Hospitalier Régional of Orléans*, France.

## Methods

### Study Design

**Fig. 1:**
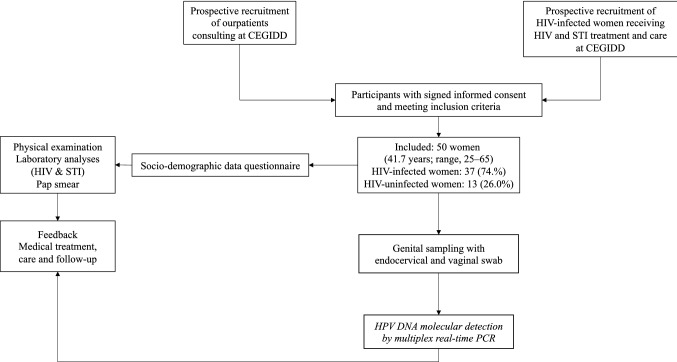
Flow diagram of the ImmiPap study. A total of 50 first-generation African immigrant women living in Orléans, France, were enrolled prospectively, including 37 (74.4%) HIV-infected women followed-up for HIV infection and STIs at the *Centre Gratuit d’Information, de Dépistage et de Diagnostic* (CeGIDD) of Orléans, France, and the *Centre Hospitalier Régional d'Orléans* and 13 (26.0%) HIV-uninfected women from general population consulting the CeGIDD for vaccination and STIs screening, prevention, prophylaxis, and care. After completing the socio-demographic data questionnaire, the included women underwent cervicovaginal swabbing for HPV molecular analyses and genital sampling for Pap smear test. Venipuncture blood sample was obtained from each woman for HIV and STIs screening. Finally, feedback on results was returned to each women and medical treatment, care and follow-up was given appropriately if needed

The study was a descriptive cross-sectional survey to assess molecular testing for cervical HPV and cervical cytology among HIV-infected and uninfected first-generation immigrant African women (at least 25 years) attending the *Centre Hospitalier Régional d'Orléans* (Fig. 1). This study was especially focused on immigrant women born in sub-Saharan Africa and currently living in France and who had never had molecular or cytological screening for cervical HPV infection and precancerous lesions before migration and since their arrival in France.

No data is available concerning HPV prevalence in African immigrant women living in France. To estimate the minimum sample size for this study we have used the minimum HPV prevalence reported for immigrant women living in Italy [14], a neighboring country of France with sub-Saharan immigration. By setting a minimum 80% statistical power, and considering an expected HPV prevalence for immigrant women living in Italy of at least 7.8% [14], an estimated convenience sample size of at least 48 subjects was obtained using the Cochran's formula modified for sample size calculation in small populations (EpiInfo, version 3.5.4).

### Enrolment, Selection Criteria and Study Population

First-generation immigrant African women attending the *Centre Gratuit d’Information, de Dépistage et de Diagnostic* (CeGIDD) of the *Centre Hospitalier Régional d'Orléans* were prospectively included in 2018. The CeGIDD is an outpatient consultation service providing HIV, STIs, hepatitis and HPV prevention, screening and care for the general adult population as well as HIV-treatment and medical follow-up for HIV-infected individuals. The CeGIDD is regularly attended by women of various origins wishing to be screened and cared for infectious diseases, mainly STIs, and also to have contraception. Furthermore, primary molecular testing for HPV screening is systematically offered to all women aged between 25 to 65 years attending CeGIDD centers [15]. The CeGIDD allows fast access to screening and treatment.

The main inclusion criteria were originating from sub-Saharan Africa and, never having undergone molecular or cytological screening for HPV infection and precancerous lesions. The included women have signed informed consent, were aged between 25 and 65 years, were sexually active, had no genital troubles at physical examination and had completed the study questionnaire. Exclusion criteria included age less than 25 years and more than 65 years, having previously undergone molecular or cytological screening for cervical HPV infection and precancerous lesions since the arrival in France, not willing to participate to the study or to answer the face-to-face sociodemographic and medical data questionnaire. All women from general population reaching these inclusion criteria were included in the study. Knowing that our inclusion criteria were particularly specific and selective, the recruitment of women was extended to African immigrant women followed-up at the *Centre Hospitalier Régional d'Orléans* for known HIV infection.

After signed the informed consent form, the included women benefited from free STIs screening including HIV, hepatitis B (HBV) and hepatitis C (HCV) viruses, *Chlamydia trachomatis*, *Neisseria gonorrhoeae*, genital herpes and *Trichomonas vaginalis*. All women received an information session on HIV, STIs and cervical cancer, and completed a face-to-face questionnaire that included socio-demographic characteristics and behavioral data such as age, marital status, social occupation, education level, past-history of STIs, HIV status and also sexual behavioral characteristics such as the number of lifetime sexual partners, frequency of condom use and the age at first sexual intercourse. Women diagnosed positive for any STI received adequate care and those negative for HBV were vaccinated against HBV.

### Samples and Processing

After completing the medical and socio-demographic data collection questionnaire, a nurse performed cervicovaginal sampling using a flocked swab (Copan Diagnostic Inc., California, USA), frozen at − 80 °C. Cervicovaginal swab were transported in frozen ice packs, to the virology laboratory of the *Hôpital européen Georges Pompidou*, Paris, France, for molecular analyses.

After the sample procedure, included women were referred to the gynecologist to undergo Pap smear cytology. Cytological results were classified according to the Bethesda system 2001 [16]. Samples were classified as atypical squamous cell of undetermined significance (ASCUS), low-grade squamous intraepithelial lesions, high-grade squamous intraepithelial lesions, adenocarcinoma in situ and carcinoma. Cytology was evaluated by cytoscreeners and pathologists blinded to the outcomes of HPV testing.

### HPV Detection and Genotyping

HPV detection and genotyping were assessed using the real-time PCR assay Anyplex™ II HPV28 (Seegene, Seoul, South Korea), as described previously [3].

### Statistical Analyses

Means and standard deviations (SD) were calculated for quantitative variables and proportions for categorical variables. The results were presented as a 95% confidence interval (CI). Pearson’s χ^2^ or Fisher's exact tests were used for categorical variables and the non-parametric Mann–Whitney *U*-test for quantitative variables.

### Ethics Statement

The Scientific Committee of the Centre *Hospitalier Régional d’Orléans* formally approved the study. All included women gave their informed signed consent to participate to the study. For each included woman, the record of the consent to participate in the study was documented on each questionnaire. The Ethical Committee formally approved this consent procedure. Feedback of medical results and particularly all results of HPV detection and genotyping were given to each participant. Women infected with any STI, those harboring cervical HR-HPV and women with HPV-related lesions received adequate treatment and therapeutic follow up.

## Results

### Characteristics of Study Participants

A total of 50 women (mean age, 41.7 years; range, 25–65) living in Orléans, France, for an average of 10.7 years (range, 1–32) were prospectively included (Table 1). HIV-infected African immigrant women (37/50; 74.0%) followed-up at the CeGIDD were more represented than HIV-negative African immigrant from general population (26.0%; 13/50).

**Table 1:** Baseline characteristics of 50 African immigrant women living in Orléans, France

	Study women (N = 50)
Characteristics	n (%) [95% CI]^a^
Age	
All age [mean (SD), years]	41.7 (10.3)
25–29	5 (10.0) [1.7–18.3]
30–39	18 (36.0) [22.7–49.3]
40–49	18 (36.0) [22.7–49.3]
≥ 50	9 (18.0) [7.3–28.6]
HIV infection	37 (74.0) [61.8–86.2]
Marital status	
Single	23 (46.0) [32.2–59.8]
Living in couple	19 (38.0) [24.5–1.4]
Divorced	7 (14.0) [4.3–23.6]
Widowed	1 (2.0) [0.0–5.8]
Times since the entry in France (Years)	
< 5	15 (30.0) [17.3–42.7]
5–10	11 (22.0) [10.5–33.5]
11–20	20 (40.0) [26.4–53.6]
> 20	4 (8.0) [0.5–15.5]
Occupation	
Student	3 (6.0) [0.0–12.6]
Unemployed	29 (58.0) [44.3–71.7]
Employed	18 (36.0) [22.7–49.3]
Sexual behaviors	
Age at sexual onset [years]	
< 16	6 (12.0) [2.9–21.1]
16–20	37 (74.0) [61.8–86.2]
> 20	7 (14.0) [4.34–23.6]
Number of sexual partners in the last 12 months	
No partner	15 (30.0) [17.3–42.7]
One regular partner	34 (68.0) [55.1–80.9]
Several partners [1–5]	1 (2.0) [0.0–5.8]
Frequency of condom use	
Always	6 (12.0) [2.9–21.1]
Inconsistently	30 (60.0) [46.4–73.6]
Never	14 (28.0) [15.5–40.4]
Cytological cervical results	
Normal cytology	42 (84.0) [73.8–94.2]
Low grade lesion	3 (6.0) [0.0–12.6]
High grade lesion	2 (4.0) [0.0–9.4]
ASCUS	3 (6.0) [0.0–12.6]
HPV DNA detection and types	
HPV DNA in swab	34 (68.0) [55.1–80.9]
Multiple types of any HPV	12 (24.0) [12.2–35.8]
LR–HPV	15 (30.0) [17.3–42.7]
Possibly oncogenic HPV	4 (8.0) [0.5–15.5]
HR-HPV	28 (56.0) [42.2–69.7]
Multiple types of HR-HPV	7 (14.0) [4.3–23.6]
Any 9-valent vaccine types^b^	20 (40.0) [26.4–53.6]
Multiple 9-valent vaccine types	3 (6.0) [0.0–12.6]
9-valent vaccine HR-HPV types	19 (38.0) [24.5–1.4]
9-valent vaccine HR-HPV types only	12 (24.0) [12.2–35.8]
Non-vaccine HR-HPV types only	10 (20.0) [8.9–31.1]

*95% CI* 95% confidence interval; *HIV* human immunodeficiency virus; *HPV* human papillomavirus; *LR-HPV* low-risk human papillomavirus; *HR-HPV* high-risk human papillomavirus

^a^The frequency of each variable is presented with their 95% confidence interval in brackets

^b^The 9-valent Gardasil-9® vaccine (Merck & Co. Inc.) is effective against HPV genotypes 6, 11, 16, 18, 31, 33, 45, 52 and 58

Single women (46.0%) were a little more represented than women living in life-couple with a male partner (38.0%). Although the majority of these African immigrant women were unemployed (58.0%), a relatively high number of them (36.0%) reported having a regular paid job in France, while only 3 women (6.0%) were students. Sexual intercourse onset occurred generally (74.0%) between 16 and 20 years. The majority (68.0%) of study women had one regular sexual partner during the last 12 months. Unprotected sexual intercourse was particularly frequent, as only 12.0% of study participants declared to systematically use condom during sex, while the large majority (60.0%) of them reported condom use inconsistently during sex. Unexpectedly, 28.0% of participants reported never using condom with their sexual partner.

Finally, two (4.0%) women declared having had previous acute genital herpes and another one (2.0%) reported past history of genital *Chlamydia* infection. No past history of any other STIs such as syphilis, gonorrhea and genital condyloma was declared. In addition, none of the women included in the study had been vaccinated against HPV infection and had ever undergone molecular and cytological screening for HPV infection and precancerous lesions, both in their country of origin, and also since their arrival in France.

Table 2 depicts the characteristics of study women according to HIV serostatus. HIV-positive women reported more frequently never using condom during sex than HIV-negative women (P < 0.05). Among study women, no student was found to be infected with HIV despite the fact that all of them reported condom use only occasionally. Finally, no other statistically significant difference could be found between HIV-infected and HIV-uninfected women in this study.

**Table 2:** Characteristics of the study women according to their HIV serostatus

	HIV-positive women (N = 37) n (%) [95% CI]*	HIV-negative women (N = 13) n (%) [95% CI]*	P**
Characteristics			
Age			
All age [mean (SD), years]	42.8 (9.7)	38.6 (11.6)	0.17
25–29	2 (5.4) [0.0–12.7]	3 (23.1) [0.2–45.9]	0.10
30–39	13 (35.1) [19.7–50.5]	5 (38.4) [12.1–64.9]	1
40–49	16 (43.2) [27.3–59.2]	2 (15.4) [0.0–35.0]	0.09
≥ 50	6 (16.2) [4.3–28.1]	3 (23.1) [0.2–45.9]	1
Marital status			
Single	14 (37.8) [22.2–53.5]	9 (69.2) [44.1–94.3]	0.06
Living in couple	16 (43.2) [27.3–59.2]	3 (23.1) [0.2–45.9]	0.32
Divorced	6 (16.2) [4.3–28.1]	1 (7.7) [0.0–22.2]	0.65
Widowed	1 (2.7) [0.0–7.9]	0 (0.0) [0.0–0.0]	1
Times since the entry in France (Years)			
< 5	10 (27.1) [12.7–41.3]	5 (38.4) [12.1–64.9]	0.49
5–10	8 (21.6) [8.4–34.9]	3 (23.1) [0.2–45.9]	1
11–20	16 (43.2) [27.3–59.2]	4 (30.7) [5.7–55.8]	0.52
> 20	3 (8.1) [0.0–16.9]	1 (7.7) [0.0–22.2]	1
Occupation			
Student	0 (0.0) [0.0–0.0]	3 (23.1) [0.2–45.9]	0.014
Unemployed	21 (56.7) [40.8–72.7]	8 (61.5) [35.1–87.9]	1
Employed	16 (43.2) [27.3–59.2]	2 (15.4) [0.0–35.0]	0.09
Sexual behaviors			
Age at sexual onset [years]			
< 16	6 (16.2) [4.3–28.1]	0 (0.0) [0.0–0.0]	0.32
16–20	28 (75.7) [61.8–89.5]	9 (69.2) [44.1–94.3]	0.72
> 20	3 (8.1) [0.0–16.9]	4 (30.7) [5.7–55.8]	0.06
Number of sexual partners in the last 12 months			
No partner	12 (32.4) [17.3–47.5]	3 (23.1) [0.2–45.9]	0.73
One regular partner	24 (64.8) [49.5–80.2]	10 (76.9) [54.1–99.8]	0.51
Several partners [1 to 5]	2 (5.4) [0.0–12.7]	0 (0.0) [0.0–0.0]	1
Frequency of condom use			
Always	6 (16.2) [4.3–28.1]	0 (0.0) [0.0–0.0]	0.32
Inconsistently	17 (45.9) [29.8–62.0]	13 (100.0) [100.0–100.0]	0.0005
Never	14 (37.8) [22.2–53.5]	0 (0.0) [0.0–0.0]	0.01
Cytological cervical results			
Normal cytology	30 (81.1) [68.4–93.7]	12 (92.3) [77.8–100.0]	0.66
Low grade lesion	3 (8.1) [0.0–16.9]	0 (0.0) [0.0–0.0]	0.55
High grade lesion	2 (5.4) [0.0–12.7]	0 (0.0) [0.0–0.0]	1
ASCUS	2 (5.4) [0.0–12.7]	1 (7.7) [0.0–22.2]	1
HPV DNA detection and types			
HPV DNA in swab	26 (70.3) [55.5–85.0]	8 (61.5) [35.1–87.9]	0.73
Multiple types of any HPV	11 (29.7) [15.0–44.4]	1 (7.7) [0.0–22.2]	0.14
LR-HPV	13 (35.1) [19.7–50.5]	2 (15.4) [0.0–35.0]	0.29
Possibly oncogenic HPV	4 (10.8) [0.8–20.8]	0 (0.0) [0.0–0.0]	0.56
HR-HPV	23 (62.2) [46.5–77.8]	5 (38.4) [12.1–64.9]	0.19
Multiple types of HR-HPV	6 (16.2) [4.3–28.1]	1 (7.7) [0.0–22.2]	0.66
Any 9-valent vaccine types**	16 (43.2) [27.3–59.2]	4 (30.7) [5.7–55.8]	0.52
Multiple 9-valent vaccine types	3 (8.1) [0.0–16.9]	0 (0.0) [0.0–0.0]	0.55
9-valent vaccine HR-HPV types	16 (43.2) [27.3–59.2]	3 (23.1) [0.2–45.9]	0.32
9-valent vaccine HR-HPV types only	11 (29.7) [15.0–44.4]	1 (7.7) [0.0–22.2]	0.14
Non-vaccine HR-HPV types only	6 (16.2) [4.3–28.1]	4 (30.7) [5.7–55.8]	0.42

### Prevalence of HPV Detection, Genotype Distribution and Cytological Results

Overall, more than two-thirds of study women were shedding genital HPV DNA, corresponding to a prevalence of HPV detection of 68.0% (34/50), with 56.0% (28/50) of HR-HPV genotypes (Table 1). Genital infection profiles with multiple HPV genotypes were relatively frequent (24.0%), with an average of 2.1 h-HPV (range, 1–4) per cervical swab sample.

HIV-infected women showed a trend to be more frequently infected by HPV than HIV-negative women, but without statistical significance (70.3% *versus* 61.5%; P = 0.73). Similarly, HIV-infected women were almost twice more infected by HR-HPV than HIV-negative women, but the difference was not significant (62.2% *versus* 38.4%; P = 0.19). However, the prevalence of HPV-68 was higher in HIV-uninfected women (23.1%, 3/13) than in HIV-infected women (18.9%, 7/37), but this difference was not statistically significant (P = 0.70).

**Fig. 2:**
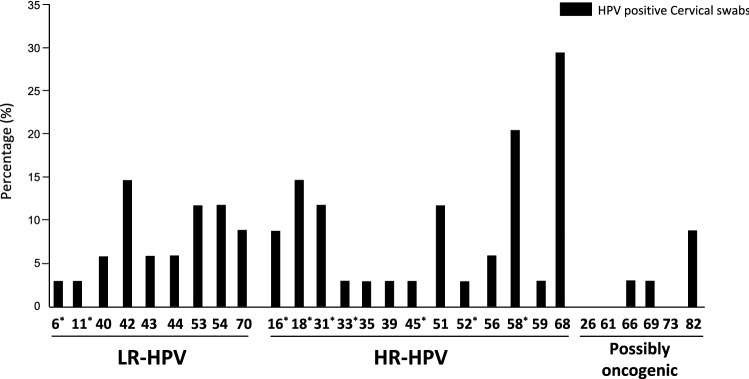
Proportion of cervical HPV genotypes according to their inclusion in the 9-valent Gardasil-9® vaccine. Number of low-risk (LR), high-risk (HR) and possibly oncogenic HPV genotypes in 34 cervical specimens positive for HPV DNA by molecular biology according to their possible prevention by 9-valent Gardasil-9® HPV vaccine among adult first-generation immigrant women originating from sub-Saharan Africa and living in France. The Anyplex™ II HPV28 kit (Seegene) allows to distinguish 28 HPV genotypes, including 13 high-risk types (HR-HPV -16, -18, -31, -33, -35, -39, -45, -51, -52, -56, -58, -59, and -68), 9 low-risk (LR) types (LR-HPV -6, -11, -40, -42, -43, -44,-53, -54 and -70) and then, 6 genotypes classified as possibly carcinogenic (HPV-26, -61, -66, -69, -73 and -82) [3]. Study women were mainly originating from Central Africa, including Democratic Republic of Congo (32.0%), Cameroon (14.0%), Central African Republic (10.0%), Angola (6.0%), Rwanda (6.0%), Burundi (2%) and Gabon (2%), while the remaining women were from West Africa, including Ivory Coast (10.0%), Nigeria (4%), and other west-African countries (Guinea, Mali, Mauritania, Senegal and Togo; 2%). One (2.0%) participant was from Ethiopia. Nota bene. The 9-valent Gardasil-9® vaccine (Merck & Co. Inc., New Jersey, USA) is effective against HPV genotypes -6, -11, -16, -18, -31, -33, -45, -52 and -58, as indicated by stars

Figure 2 depicts the atypical profile in the distribution of HPV genotypes in HPV-DNA positive cervical samples, with the non-vaccine HR-HPV-68 (20.0%) being the predominant genotype, followed by the Gardasil-9® vaccine HR-HPV-58 with a prevalence of 14.0%. HR-HPV-16 and HR-HPV-18 were respectively detected in 6.0% and 10.0% of cervical swabs. Apart from the HPV-31 found in 8.0% of the women, the other Gardasil-9® vaccine types (HPV-6, HPV-11, HPV-33, HPV-45 and HPV-52) were each detected only in one (2.0%) woman. Similarly, the other non-vaccine HR-HPV types (HPV-35, HPV-39, HPV-56 and HPV-59) were also represented with very low proportion.

Results of Pap smear cytological exam are shown in Table 1. The large majority of women (84.0%) showed normal cytology, but 6.0% harbored low-grade lesions and ASCUS, and only two women (4.0%) exhibited high-grade cervical lesions. Furthermore, all women with low- or high-grade cervical lesions were all infected with HIV (Table 2).

Finally, no predictive risk factor for cervical HPV infection in these women could be evidenced in univariate and multivariate analyses.

### Presumed Predictive Efficiency of Catch-Up Prophylactic Vaccination with Gardasil-9® HPV Vaccine

None of study women had received HPV prophylactic vaccination, which raises the issue of possible catch-up HPV vaccination for them. Study women could be divided in three principal categories according to the presence or the absence of HPV DNA in their genital secretions.

The first category consisted of more than half of the study women (56.0%; 28/50) and corresponded to women with genital HR-HPV infection. These women already infected with HR-HPV genotypes could be a priori poorly eligible for catch-up HPV vaccination.

The second category included women exclusively infected with low-risk genotypes (12.0%; 6/50). These women could be a priori eligible for possible catch-up prophylactic HPV vaccination against the seven high-risk genotypes targeted by the Gardasil-9® vaccine (HPV-16, -18, -31, -33, -45, -52 and -58).

Finally, the third category comprised one-third of the study women (32.0%; 16/50), without detectable genital HPV DNA. These women could be a priori likely eligible for possible catch-up prophylactic HPV vaccination.

## Discussion

We herein report unsuspected high burden of cervical HR-HPV infection in a consecutive series of non-vaccinated and never screened first-generation adult immigrant women originating from sub-Saharan Africa and living in France. Genital HPV and HR-HPV were highly frequent both in HIV-infected and uninfected African immigrant women. Unexpectedly, the non-vaccine HPV-68, a genotype rarely found within the French female population, was the most frequently detected. These findings point to the remarkable and atypical epidemiological profile of cervical HPV infection in first-generation African immigrants living in France, with a rare and non-vaccine high-risk genotype being the most frequently detected. Furthermore, despite their advanced age, nearly half of study women (44%; 22/50), including those only infected with low-risk HPV genotypes and those without detectable genital HPV DNA, could be eligible for catch-up HPV prophylactic vaccination with the nonavalent Gardasil-9® vaccine.

In these African immigrant women, we found a particularly high prevalence of cervical HPV (68.0%), frequently associated with HR-HPV genotypes (56.0%) found in 61.5% of HIV-negative and 70.3% of HIV-positive women. It is difficult to compare our observations with the few previously published studies on HPV cervical infection among immigrant women living in Europe. Moreover, the published studies are heterogeneous with each other, especially concerning the origin and selection of immigrant women (regular or clandestine), and the types of methodology used. However, some points can be debated. Firstly, such high prevalences of cervical HR-HPV in study women appear uncommon and higher than the HPV prevalences previously reported in the French female general population with cervical HR-HPV infection rates ranging from 8 to 22.8% in HIV-negative adult women with normal cytology [17–26] and 26.4% in HIV-positive women [27]. The HPV prevalences in study population were also largely higher than the low HPV prevalence of 7.8% in immigrant women reported in a large study conducted in Italy among 22,814 regular immigrant women from various origins (mostly from Eastern Europe and North Africa) [15]. Secondly, other studies reported results of the same order as ours in HIV-infected women and in foreign women. For example, the high prevalence of any cervical HR-HPV of 49.5% was reported in a large population-based survey on HIV-positive women living in Europe, including between 0 and 74% sub-Saharan African women depending on inclusion centres [28], that is similar to the high HPV prevalence we found in our study women. In Spain, the prevalence of HPV in the foreign women was 23.5%, significantly higher than in the Spanish women [29]. Thirdly, several reports conducted in Italy among immigrant women originating from sub-Saharan Africa showed high HPV prevalence rates, from 35.5% [30], 44.0% [31], 45.0% [32], 57.6% [33] to 65.4% [34] in HIV-negative women, and 57.1% [30] to 61.5% [33] in HIV-infected women. Finally, the high prevalences of HR-HPV infection found in the present study likely mirror the burden of cervical HR-HPV infection commonly reported in Sub-Saharan Africa. Indeed, quite similar to our study, cervical HR-HPV prevalences in sub-Saharan Africa can reach 46.2% in HIV-negative adult women (more than 25 years) and 79.1% in HIV-positive women [8–10]. Although comparisons may be difficult to establish, our observations point that African immigrants living in France constitute a population with a particularly high risk of HR-HPV cervical infection, irrespective of their HIV-status, being significantly more infected by HR-HPV than Western European female native population, and also probably more than immigrant women originating from Eastern Europe. Taken together, according to our own observations and those previously reported in the literature in immigrant women living in Europe and in women living in sub-Saharan Africa, the prevalences of genital HPV appear highly heterogeneous. In HIV-negative women, HPV prevalences range from 35 to 65% in immigrant adult women from sub-Saharan Africa living in Europe and from 25 to 50% in adult women living in sub-Saharan Africa. The genital carriage of HPV is furthermore clearly increased in case of HIV-1-coinfection, with prevalences as high as 60–70% in immigrant women originating from sub-Saharan Africa living in Europe and 80% in adult women living in sub-Saharan Africa.

As a consequence, adult first-generation African immigrant women living in France, especially those infected with HIV, should be at very high risk for developing cervical cancer. In Italy, a neighboring country of France, the estimated risk for high grade cervical lesions and cancers was effectively higher in immigrant women from high HPV prevalence regions, especially sub-Saharan African countries, as compared to native women living in Italy as well as in other high-income European countries [35]. However, the duration of stay in France of our study women (10 years on average) makes it difficult to clearly state whether the HPV prevalence pattern found in the present study is related to HPV exposure before migration or is associated with factors occurring during the sexual life of these women in France. Further multicenter studies with larger sample sizes are now needed to better estimate the cervical cancer burden in the population of first-generation immigrant women originating from sub-Saharan Africa and living in France.

The distribution of HPV genotypes in women, with a majority harboring the non-vaccine HPV-68, was different to that previously reported in French women born to French parents with HR-HPV-16 as the most detected genotype [24]. This unusual and original epidemiological profile of HPV infection in a subgroup of the population of adult women living in France could be explained by the social conditions of these immigrant women at their arrival in France. Indeed, immigrant women from sub-Saharan Africa living in France, especially those of first-generation, remain mostly in the same social network at their arrival in France, which is mainly constituted by people also coming from their native regions where these unusual genotypes are frequent [36].

The wide heterogeneity of the HPV genotypes distribution in our study female immigrant population was previously reported in Africa [7, 8, 10, 11]. Some genotypes frequently detected in women living in Africa, such as HPV-35 or HPV-59 [7, 8, 10, 11], were seen with low prevalence in our study population, possibly suggesting that the African epidemiological pattern could have been gradually diluted in these immigrants women living in France for an average of 10 years. Nevertheless, as for HIV epidemic in some countries, in which imported sub-epidemics may occur, characterized by HIV-subtypes and strains genetically and phenotypically different from those commonly observed [37], adult immigrant women originating from sub-Saharan Africa could also import HPV genotypes different from those commonly spreading in French women born in France. Such imported viruses could be more aggressive and phenotypically less recognizable by the immune system of vaccinated people [38]. Further genetic studies are obviously needed to better address this aspect of HPV epidemiology.

For immigrant women at risk for cervical cancer, regular HPV molecular testing, cytological screening for precancerous cervical lesions and adequate treatment remain the best suitable preventive approach to avoid the occurrence of cervical cancer [15, 39–42]. In addition, the issue of catch-up HPV prophylactic vaccination should be addressed in this population at high risk of cervical cancer. Current prophylactic HPV vaccines are thought to confer better protection against HPV infection mainly in individuals who have not been yet infected [43, 44]. HPV vaccination policies in most countries such as France recommend vaccination in girls before age of 15 (11–14 years), with a catch up until 19 years old [45, 46]. Recently, the Advisory Committee on Immunization Practices (ACIP) in the United States of America has updated their HPV vaccination guidelines in extending the age of eligibility for HPV vaccination up to 45 years old according to the virological, immunological and clinical parameters rather than the age criterion [47–49]. Possible interest of catch-up HPV prophylactic vaccination extended to adult women at high risk for HR-HPV infection and cervical cancer, could be envisioned in first-generation immigrant adult African women living in France. Indeed, prophylactic HPV vaccines have shown to provide almost 67–90% protection against the persistence of HPV infection as well as the occurrence of high-grade precancerous lesions, over 7 years after vaccination, in adult women up to 45 years old [42, 50–52], even in women with serological evidence of prior HPV exposure [53, 54]. In addition, only 20% of study women were exclusively infected by high-risk HPV genotypes not targeted by the Gardasil-9® vaccine, suggesting that at least 80% of genital HPV infections in this sample of first-generation immigrant women living in France would be a priori prevented by the vaccination with Gardasil-9® vaccine. Ongoing clinical trials such as HPV-FASTER study already suggest that extending routine vaccination strategies to women up to age 30 years in combination with greater access to primary molecular HPV screening can hasten impact on cancer incidence [55–57]. Note that catch-up HPV prophylactic vaccination could be proposed even in HIV-infected women, in whom better immunogenicity is seen when HIV viral replication is controlled, and when there is no overt immunodeficiency [58, 59].

Finally, in our series of first-generation African immigrant women living in France and who underwent cervical cancer screening for the first time, we found a large proportion (84.0%) of normal cytological results, and only a minority exhibited pathological Pap smear test, including ASCUS (6.0%) observed both in one HIV-infected and one HIV-negative woman, and also low-grade (6.0%) and high-grade (4.0%) cervical lesions only observed in HIV-positive women. Although there was no statistically difference between HIV-infected and HIV-negative women in regards to Pap smear results, our findings show that worst cervical lesions are mostly found in HIV-positive women than in HIV-negative. Further large studies are needed to have a more precise evaluation of the burden of HPV-related cervical lesions and cancers in the African immigrant women living in France, which constitute a population in which the coverage of cervical cancer screening remains very low. Indeed, in France, foreign women born to parents coming from a foreign country were more than five times more likely to have never been screened for cervical cancer in their lifetime than French women born to French parents [60]. Furthermore, recent population-based surveys reported that African immigrant population living in France is subjected to a very high risk of being denied care by health-care professionals [36, 61, 62].

### Study Limitations

Our study has some limitations. Firstly, the inclusion sites in Orléans limited to outpatient consultation services for HIV and STIs may have introduced a recruitment bias in favor of HIV- and STI- infected women. Secondly, women who consulted for the management of STI could clearly be more at risk of carrying HPV. Therefore, healthy African immigrant women are under-represented in our study. Thirdly, the high frequency of HIV-infected women in our study may have resulted in overestimating the prevalences of cervical HPV molecular detection. Fourth, the small sample size of our study population makes these results not fully representative of the first-generation immigrant women originating from sub-Saharan Africa and living in France. Finally, our conclusions on a high HPV infection rate among immigrants from sub-Saharan Africa living in France should be conditional and call for other studies on larger samples.

### New Contribution to the Literature

Our study highlights for the first time the high prevalence of cervical HPV infection with original genotypes distribution frequently associated with HIV infection in never-screened immigrant women originating from sub-Saharan Africa and living in France. There is an urgent need to implement adapted screening interventions specific for sub-Saharan African immigrant women who appear to be at very high risk for cervical cancer. Regular access to HPV molecular screening and Pap smear triage constitutes likely the necessary condition to allow preventing genital cancers in immigrant African women living in France. New strategies using genital self-sampling have proven to be as suitable for HPV molecular testing as the usual clinician-administered vaginal sampling [25, 63–65]. Furthermore, genital self-sampling is a good alternative to reach remote and neglected populations with limited access to healthcare facilities in in sub-Saharan Africa [64, 65]. Such new strategy effective for women in Africa could be adapted for first-generation African women living in France. Indeed, in a randomized trial conducted in France, the self-sampling strategy was more effective and cost-effective than the recall letter commonly used in France, increasing thus the participation in cervical cancer screening [63]. Therefore, offering at social welfare centers, as well as in hot spots of residence of immigrant populations of African origin an in-home return-mail kit vaginal self-sampling device, could help to increase the cervical cancer screening coverage in this key and neglected sub-population of French women. In addition, special attention should be also focused on HIV-infected immigrant women, since HIV constitutes an aggravating factor for the progression of HPV-related lesions to invasive cancer [66]. Finally, further investigations are necessary to better identify the social and cultural barriers of cervical cancer screening specific to the sub-Saharan African immigrant women living in France, especially those of first-generation who are most often no longer eligible for HPV prophylactic vaccination.

## Electronic Supplementary Material

Below is the link to the electronic supplementary material.

Supplementary File 1 (XLSX 32 kb)

## References

[1] Scheurer ME, Tortolero-Luna G, Adler-Storthz K (2005). Human papillomavirus infection: biology, epidemiology, and prevention. Int J Gynecol Cancer.

[2] Parkin DM (2006). The global health burden of infection-associated cancers in the year 2002. Int J Cancer.

[3] Mboumba Bouassa RS, Mbeko Simaleko M, Camengo SP, Mossoro-Kpinde CD, Veyer D, Matta M, Robin L, Longo JD, Grésenguet G, Péré H, Meye JF, Belec L (2018). Unusual and unique distribution of anal high-risk human papillomavirus (HR-HPV) among men who have sex with men living in the Central African Republic. PLoS ONE.

[4] De Vuyst H, Alemany L, Lacey C, Chibwesha CJ, Sahasrabuddhe V, Banura C, Denny L, Parham GP (2013). The burden of human papillomavirus infections and related diseases in sub-saharan Africa. Vaccine.

[5] Ferlay J, Soerjomataram I, Dikshit R, Eser S, Mathers C, Rebelo M, Parkin DM, Forman D, Bray F (2015). Cancer incidence and mortality worldwide: sources, methods and major patterns in GLOBOCAN 2012. Int J Cancer.

[6] World health Organization, 2015. Projections of mortality and causes of death, 2015 and 2030. https://www.who.int/healthinfo/global_burden_disease/projections/en/. Accessed July 2019.

[7] Mboumba Bouassa RS, Prazuck T, Lethu T, Jenabian MA, Meye JF, Bélec L (2017). Cervical cancer in sub-Saharan Africa: a preventable noncommunicable disease. Expert Rev Anti Infect Ther.

[8] Kelly HA, Sawadogo B, Chikandiwa A, Segondy M, Gilham C, Lompo O, Omar T, Didelot MN, Nagot N, Meda N, Weiss HA, Delany-Moretlwe S, Mayaud P, HARP Study Group (2017). Epidemiology of high-risk human papillomavirus and cervical lesions in African women living with HIV/AIDS: effect of anti-retroviral therapy. AIDS..

[9] Ginindza TG, Dlamini X, Almonte M, Herrero R, Jolly PE, Tsoka-Gwegweni JM, Weiderpass E, Broutet N, Sartorius B (2017). Prevalence of and associated risk factors for high risk human papillomavirus among sexually active women, Swaziland. PLoS ONE.

[10] Ogembo RK, Gona PN, Seymour AJ, Park HS, Bain PA, Maranda L, Ogembo JG (2015). Prevalence of human papillomavirus genotypes among African women with normal cervical cytology and neoplasia: a systematic review and meta-analysis. PLoS ONE.

[11] Akarolo-Anthony SN, Famooto AO, Dareng EO, Olaniyan OB, Offiong R, Wheeler CM, Adebamowo CA (2014). Age-specific prevalence of human papilloma virus infection among Nigerian women. BMC Public Health.

[12] Institut National de la Statistique et des Etudes Economiques (INSEE). IMG1B-Population immigrée par sexe, âge et pays de naissance en 2014. https://www.insee.fr/fr/statistiques/2874036?sommaire=2874056&geo=METRO-1. Accessed March 2018.

[13] McCloskey JC, Kast WM, Flexman JP, McCallum D, French MA, Phillips M (2017). Syndemic synergy of HPV and other sexually transmitted pathogens in the development of high-grade anal squamous intraepithelial lesions. Papillomavirus Res.

[14] Campari C, Fedato C, Petrelli A, Zorzi M, Cogo C, Caprioglio A, Gallo F, Giordano L, Domenighini S, Pasquale L, Prandi S, Zappa M, Giorgi Rossi P, GISCi Migrant Working Group (2015). HPV prevalence and risk of pre-cancer and cancer in regular immigrants in Italy: results from HPV DNA test-based screening pilot programs. Infect Agent Cancer..

[15] Haute autorité de santé. Dépistage Évaluation de la recherche des papillomavirus humains (HPV) en dépistage primaire des lésions précancéreuses et cancéreuses du col de l’utérus et de la place du double immunomarquage p16/Ki67. Synthèse et recommandations. P. 36. Saint-Denis La Plaine: Haute autorité de santé (HAS); juillet 2019. https://www.has-sante.fr/upload/docs/application/pdf/2019–07/synthese_et_recommandations_hpv.pdf. Accessed Sep 2019.

[16] Solomon D, Davey D, Kurman R, Moriarty A, O'Connor D, Prey M, Raab S, Sherman M, Wilbur D, Wright T Jr., Young N; Forum Group Members; Bethesda 2001 Workshop. The 2001 Bethesda System: terminology for reporting results of cervical cytology. JAMA. 2002;287(16):2114–9. 10.1001/jama.287.16.2114 11966386

[17] Riethmuller D, Gay C, Bertrand X, Bettinger D, Schaal JP, Carbillet JP, Lassabe C, Arveux P, Seilles E, Mougin C (1999). Genital human papillomavirus infection among women recruited for routine cervical cancer screening or for colposcopy determined by Hybrid Capture II and polymerase chain reaction. Diagn Mol Pathol.

[18] Clavel C, Masure M, Bory JP, Putaud I, Mangeonjean C, Lorenzato M, Nazeyrollas P, Gabriel R, Quereux C, Birembaut P (2001). Human papillomavirus testing in primary screening for the detection of high-grade cervical lesions: a study of 7932 women. Br J Cancer.

[19] Boulanger JC, Sevestre H, Bauville E, Ghighi C, Harlicot JP, Gondry J (2004). Epidemiology of HPV infection. Gynecol Obstet Fertil.

[20] Pannier-Stockman C, Segard C, Bennamar S, Gondry J, Boulanger JC, Sevestre H, Castelain S, Duverlie G (2008). Prevalence of HPV genotypes determined by PCR and DNA sequencing in cervical specimens from French women with or without abnormalities. J Clin Virol.

[21] Bruni L, Diaz M, Castellsagué X, Ferrer E, Bosch FX, de Sanjosé S (2010). Cervical human papillomavirus prevalence in 5 continents: meta-analysis of 1 million women with normal cytological findings. J Infect Dis.

[22] Monsonego J, Hudgens MG, Zerat L, Zerat JC, Syrjänen K, Halfon P, Ruiz F, Smith JS (2011). Evaluation of oncogenic human papillomavirus RNA and DNA tests with liquid-based cytology in primary cervical cancer screening: the FASE study. Int J Cancer.

[23] Vaucel E, Coste-Burel M, Laboisse C, Dahlab A, Lopes P (2011). Human papillomavirus genotype distribution in cervical samples collected in routine clinical practice at the Nantes University Hospital. France Arch Gynecol Obstet.

[24] Heard I, Tondeur L, Arowas L, Falguières M, Demazoin MC, Favre M (2013). Human papillomavirus types distribution in organised cervical cancer screening in France. PLoS ONE.

[25] Haguenoer K, Giraudeau B, Gaudy-Graffin C, de Pinieux I, Dubois F, Trignol-Viguier N, Viguier J, Marret H, Goudeau A (2014). Accuracy of dry vaginal self-sampling for detecting high-risk human papillomavirus infection in cervical cancer screening: a cross-sectional study. Gynecol Oncol.

[26] Bruni L, Albero G, Serrano B, Mena M, Gómez D, Muñoz J, Bosch FX, de Sanjosé S. ICO/IARC Information Centre on HPV and Cancer (HPV Information Centre). Human Papillomavirus and Related Diseases in France. Summary Report 10 December 2018. https://www.hpvcentre.net/statistics/reports/FRA.pdf

[27] Heard I, Etienney I, Potard V, Poizot-Martin I, Moore C, Lesage AC, Ressiot E, Crenn-Hebert C, Fléjou JF, Cubie H, Costagliola D, Darragh TM, ANRS-C017 VIHGY Study Group (2015). High prevalence of anal human papillomavirus-associated cancer precursors in a contemporary cohort of asymptomatic HIV-infected women. Clin Infect Dis..

[28] Heard I, Cubie HA, Mesher D, Sasieni P, MACH-1 Study Group (2013). Characteristics of HPV infection over time in European women who are HIV-1 positive. BJOG.

[29] García-Villanueva S, Domínguez-Gil González M, Gayete Martínez J, Muñoz Bellido JL, Salas Valien JS, EchevarriaIturbe C, González Sagrado M, Jiménez Pérez JM, Curiel de Arcaute López A, Rojo Rello S, Eiros Bouza JM, Ortiz de Lejarazu Leonardo R (2019). Comparative study of the prevalence of the human papilloma virus in Spanish and foreign women participating in a population screening programme in Castilla y León. Enferm Infecc Microbiol Clin..

[30] Tornesello ML, Duraturo ML, Buonaguro L, Vallefuoco G, Piccoli R, Palmieri S, Buonaguro FM (2007). Prevalence of human papillomavirus genotypes and their variants in high risk West Africa women immigrants in South Italy. Infect Agent Cancer.

[31] Tornesello ML, GiorgiRossi P, Buonaguro L, Buonaguro FM, HPV Prevalence Italian Working Group (2014). Human Papillomavirus infection and cervical neoplasia among migrant women living in Italy. Front Oncol..

[32] Giovannelli L, Vassallo R, Matranga D, Affronti M, Caleca MP, Bellavia C, Perino A, Ammatuna P (2009). Prevalence of cervical human papillomavirus infection and types among women immigrated to Sicily, Italy. Acta Obstet Gynecol Scand.

[33] Tornesello ML, Cassese R, De Rosa N, Buonaguro L, Masucci A, Vallefuoco G, Palmieri S, Schiavone V, Piccoli R, Buonaguro FM (2011). High prevalence of human papillomavirus infection in Eastern European and West African women immigrants in South Italy. APMIS.

[34] Paba P, Morosetti G, Criscuolo AA, Chiusuri V, Marcuccilli F, Sesti F, Piccione E, Perno CF, Ciotti M (2012). Prevalence of human papillomavirus infection in Italian and immigrant women. Int J Immunopathol Pharmacol..

[35] Di Felice E, Caroli S, Paterlini L, Campari C, Prandi S, Giorgi RP (2015). Cervical cancer epidemiology in foreign women in Northern Italy: role of human papillomavirus prevalence in country of origin. Eur J Cancer Prev.

[36] Pannetier J, Ravalihasy A, Lydié N, Lert F, Desgrées du Loû A, Parcours Study Group (2018). Prevalence and circumstances of forced sex and post-migration HIV acquisition in sub-Saharan African migrant women in France: an analysis of the ANRS-PARCOURS retrospective population-based study. Lancet Public Health..

[37] Vermund SH, Leigh-Brown AJ (2012). The HIV Epidemic: High-Income Countries. Cold Spring Harb Perspect Med.

[38] Sun M, Gao L, Liu Y, Zhao Y, Wang X, Pan Y, Ning T, Cai H, Yang H, Zhai W, Ke Y (2012). Whole genome sequencing and evolutionary analysis of human papillomavirus type 16 in central China. PLoS ONE.

[39] World Health Organization. Human papillomavirus vaccines. WHO position paper. Releve epidemiologique hebdomadaire / Section d'hygiene du Secretariat de la Societe des Nations = Weekly epidemiological record / Health Section of the Secretariat of the League of Nations. 2009;84(15):118–31. 19360985

[40] World Health Organization. 2013 World health organization. Global action plan for the prevention and control of noncommunicable diseases 2013–2030. 2013. https://apps.who.int/iris/bitstream/10665/94384/1/9789241506236_eng.pdf?ua=1. Accessed Sep 2019.

[41] Haute autorité de santé. Dépistage et prévention du cancer du col de l’utérus: actualisation du référentiel de pratiques de l’examen périodique de santé (EPS). Saint-Denis La Plaine: Haute autorité de santé (HAS); juin 2013. p. 55. https://www.has-sante.fr/upload/docs/application/pdf/2013-08/referentieleps_format2clic_kc_col_uterus_2013-30-08__vf_mel.pdf. Accessed Sep 2019.

[42] Wheeler CM, Kjaer SK, Sigurdsson K, Iversen OE, Hernandez-Avila M, Perez G, Brown DR, Koutsky LA, Tay EH, García P, Ault KA, Garland SM, Leodolter S, Olsson SE, Tang GW, Ferris DG, Paavonen J, Steben M, Bosch FX, Dillner J, Joura EA, Kurman RJ, Majewski S, Muñoz N, Myers ER, Villa LL, Taddeo FJ, Roberts C, Tadesse A, Bryan J, Lupinacci LC, Giacoletti KE, James M, Vuocolo S, Hesley TM, Barr E (2009). The impact of quadrivalent human papillomavirus (HPV; types 6, 11, 16, and 18) L1 virus-like particle vaccine on infection and disease due to oncogenic nonvaccine HPV types in sexually active women aged 16–26 years. J Infect Dis.

[43] Harper DM, DeMars LR (2017). HPV vaccines—a review of the first decade. Gynecol Oncol.

[44] Pinto LA, Dillner J, Beddows S, Unger ER (2018). Immunogenicity of HPV prophylactic vaccines: serology assays and their use in HPV vaccine evaluation and development. Vaccine..

[45] World Health Organization. 2017. Human papillomavirus vaccines: WHO position paper, May 2017/Vaccins contre les papillomavirus humains : note de synthèse de l’OMS, mai 2017. Weekly Epidemiological Record = Relevé épidémiologique hebdomadaire, 92(19);241–268. World Health Organization = Organisation mondiale de la Santé. https://apps.who.int/iris/handle/10665/255354. Accessed Sep 2019. 28530369

[46] Haut Conseil de la santé publique. [Avis du 28/09/2012 relatif à la révision de l’âge de vaccination contre les infections à papillomavirus humains des jeunes filles]. September 2012. https://www.hcsp.fr/Explore.cgi/avisrapportsdomaine?clefr=302. Accessed March 2019.

[47] Food and Drug Administration. Prescribing information [package insert]. Gardasil 9 (human papillomavirus 9-valent vaccine, recombinant). Silver Spring, MD: US Department of Health and Human Services, Food and Drug Administration; 2018. https://www.fda.gov/media/90064/download

[48] Levy B, Downs LS (2019). The HPV vaccine is now recommended for adults aged 27–45: counselling implications. OBG Manag.

[49] Meites E, Szilagyi PG, Chesson HW, Unger ER, Romero JR, Markowitz LE (2019). Human papillomavirus vaccination for adults: updated recommendations of the advisory committee on immunization practices. MMWR Morb Mortal Wkly Rep.

[50] Einstein MH, Levin MJ, Chatterjee A, Chakhtoura N, Takacs P, Catteau G, Dessy FJ, Moris P, Lin L, Struyf F, Dubin G, HPV-010 Study Group (2014). Comparative humoral and cellular immunogenicity and safety of human papillomavirus (HPV)-16/18 AS04-adjuvanted vaccine and HPV-6/11/16/18 vaccine in healthy women aged 18–45 years: follow-up through Month 48 in a Phase III randomized study. Hum Vaccin Immunother..

[51] Skinner SR, Szarewski A, Romanowski B, Garland SM, Lazcano-Ponce E, Salmerón J, Del Rosario-Raymundo MR, Verheijen RH, Quek SC, da Silva DP, Kitchener H, Fong KL, Bouchard C, Money DM, Ilancheran A, Cruickshank ME, Levin MJ, Chatterjee A, Stapleton JT, Martens M, Quint W, David MP, Meric D, Hardt K, Descamps D, Geeraerts B, Struyf F, Dubin G; VIVIANE Study Group. Efficacy, safety, and immunogenicity of the human papillomavirus 16/18 AS04-adjuvanted vaccine in women older than 25 years: 4-year interim follow-up of the phase 3, double-blind, randomised controlled VIVIANE study. Lancet. 2014;384(9961):2213–27. 10.1016/S0140-6736(14)60920-X 25189358

[52] Wheeler CM, Skinner SR, Del Rosario-Raymundo MR, Garland SM, Chatterjee A, Lazcano-Ponce E, Salmerón J, McNeil S, Stapleton JT, Bouchard C, Martens MG, Money DM, Quek SC, Romanowski B, Vallejos CS, Ter Harmsel B, Prilepskaya V, Fong KL, Kitchener H, Minkina G, Lim YKT, Stoney T, Chakhtoura N, Cruickshank ME, Savicheva A, da Silva DP, Ferguson M, Molijn AC, Quint WGV, Hardt K, Descamps D, Suryakiran PV, Karkada N, Geeraerts B, Dubin G, Struyf F; VIVIANE Study Group. Efficacy, safety, and immunogenicity of the human papillomavirus 16/18 AS04-adjuvanted vaccine in women older than 25 years: 7-year follow-up of the phase 3, double-blind, randomised controlled VIVIANE study. Lancet Infect Dis. 2016;16(10):1154–1168. 10.1016/S1473-3099(16)30120-7 27373900

[53] Olsson SE, Kjaer SK, Sigurdsson K, Iversen OE, Hernandez-Avila M, Wheeler CM, Perez G, Brown DR, Koutsky LA, Tay EH, García P, Ault KA, Garland SM, Leodolter S, Tang GW, Ferris DG, Paavonen J, Lehtinen M, Steben M, Bosch FX, Dillner J, Joura EA, Majewski S, Muñoz N, Myers ER, Villa LL, Taddeo FJ, Roberts C, Tadesse A, Bryan J, Maansson R, Vuocolo S, Hesley TM, Saah A, Barr E, Haupt RM (2009). Evaluation of quadrivalent HPV 6/11/16/18 vaccine efficacy against cervical and anogenital disease in subjects with serological evidence of prior vaccine type HPV infection. Hum Vaccin.

[54] Castellsagué X, Muñoz N, Pitisuttithum P, Ferris D, Monsonego J, Ault K, Luna J, Myers E, Mallary S, Bautista OM, Bryan J, Vuocolo S, Haupt RM, Saah A (2011). End-of-study safety, immunogenicity, and efficacy of quadrivalent HPV (types 6, 11, 16, 18) recombinant vaccine in adult women 24–45 years of age. Br J Cancer.

[55] Bosch FX, Robles C, Díaz M, Arbyn M, Baussano I, Clavel C, Ronco G, Dillner J, Lehtinen M, Petry KU, Poljak M, Kjaer SK, Meijer CJ, Garland SM, Salmerón J, Castellsagué X, Bruni L, de Sanjosé S, Cuzick J (2016). HPV-FASTER: broadening the scope for prevention of HPV-related cancer. Nat Rev Clin Oncol.

[56] Bosch FX, Robles C (2018). HPV-FASTER: Combined strategies of HPV vaccination and HPV screening towards a one visit for cervical cancer preventive campaigns. Salud Publica Mex..

[57] Stern PL, Roden RB (2019). Opportunities to improve immune-based prevention of HPV-associated cancers. Papillomavirus Res.

[58] Faust H, Toft L, Sehr P, Müller M, Bonde J, Forslund O, Østergaard L, Tolstrup M, Dillner J (2016). Human Papillomavirus neutralizing and cross-reactive antibodies induced in HIV-positive subjects after vaccination with quadrivalent and bivalent HPV vaccines. Vaccine.

[59] Lacey CJ (2019). HPV vaccination in HIV infection. Papillomavirus Res.

[60] Grillo F, Soler M, Chauvin P. [Absence of cervical cancer screening for women in Paris metropolitan area according to their migration characteristics in 2010]. BEH 2–3–4 / 17 janvier 2012. https://opac.invs.sante.fr/doc_num.php?explnum_id=7839. Accessed March 2019.

[61] Vignier N, Dray Spira R, Pannetier J, Ravalihasy A, Gosselin A, Lert F, Lydie N, Bouchaud O, Desgrees Du Lou A, Chauvin P, PARCOURS Study Group (2018). Refusal to provide healthcare to sub-Saharan migrants in France: a comparison according to their HIV and HBV status. Eur J Public Health..

[62] Vignier N, Desgrées du Loû A, Pannetier J, Ravalihasy A, Gosselin A, Lert F, Lydié N, Bouchaud O, Dray Spira R, PARCOURS Study Group (2018). Access to health insurance coverage among sub-Saharan African migrants living in France: Results of the ANRS-PARCOURS study. PLoS ONE.

[63] Haguenoer K, Sengchanh S, Gaudy-Graffin C, Boyard J, Fontenay R, Marret H, Goudeau A, Pigneaux de Laroche N, Rusch E, Giraudeau B (2014). Vaginal self-sampling is a cost-effective way to increase participation in a cervical cancer screening programme: a randomised trial. Br J Cancer.

[64] Viviano M, Tran PL, Kenfack B, Catarino R, Akaaboune M, Temogne L, Foguem ET, Vassilakos P, Petignat P (2018). Self- versus physician-collected samples for the follow-up of human papillomavirus-positive women in sub-Saharan Africa. Int J Women’s Health.

[65] Bélec L (2019). Acceptability and accuracy of cervical cancer screening using a self-collected veil for HPV DNA testing by multiplex real-time PCR among adult women in sub-Saharan Africa. J Clin Res Med.

[66] Liu G, Sharma M, Tan N, Barnabas RV (2018). HIV-positive women have higher risk of human papilloma virus infection, precancerous lesions, and cervical cancer. AIDS.

